# Surface electromyographic activity of trunk muscles during trunk control exercises for people after stroke; effect of a mobile and stable seat for rehabilitation

**DOI:** 10.1371/journal.pone.0272382

**Published:** 2022-07-29

**Authors:** Michelle C. Haas, Bettina B. Sommer, Samuel Karrer, Matthias Jörger, Eveline S. Graf, Martin Huber, Daniel Baumgartner, Jens Bansi, Jan Kool, Christoph M. Bauer

**Affiliations:** 1 School of Health Sciences, Zurich University of Applied Sciences, Winterthur, Switzerland; 2 School of Engineering, Zurich University of Applied Sciences, Winterthur, Switzerland; 3 Physiotherapy Department, Valens Rehabilitation Centre, Valens, Switzerland; Emory University, UNITED STATES

## Abstract

The aim of this study was to explore differences in trunk muscle activity on a stable and mobile seat for people after stroke and healthy participants. Trunk control exercises are known to have a beneficial effect on trunk control, balance, and mobility after stroke. The effect of such exercises could be enhanced by the use of a mobile seat to provide further training stimuli. However, little research on the musculoskeletal effects of trunk training on mobile seats has been carried out. On a stable and a mobile seat, thirteen people after stroke and fifteen healthy participants performed two selective trunk control exercises, which were lateral flexion initiated by the pelvis and the thorax. The maximal surface electromyography relative to static sitting of the muscles multifidus, erector spinae, and obliquus externus was recorded bilaterally. The effects of group, seat condition, trunk control exercise, and muscle side were investigated employing within-subject linear-mixed-models. Compared to the stable seat, the maximal muscle activity of people after stroke on the mobile seat was higher during the thorax-initiated exercise and lower during the pelvis-initiated exercise. Healthy participants showed opposite results with higher muscle activity on the mobile seat during the pelvis-initiated exercise. For trunk control training on a mobile seat with high muscle activation people after stroke should perform trunk control exercises initiated by the thorax, for training with lower muscle activity people after stroke should initiate selective trunk movements by the pelvis. The results can support the planning of progressive trunk control rehabilitation programs.

## Introduction

The combined third-leading cause of death and disability in 2019 was stroke [1]. Therefore, it is still one of the leading causes of disease burden globally [2, 3]. While incidence rates are plateauing or decreasing slightly in richer countries, rates are increasing in poorer countries. Despite age-standardized rates of stroke having decreased over the last decade, the decline has been insufficient to compensate for world population ageing and growth. Hence, an increase is predicted for the coming years [1]. This increase additionally leads to more costs for rehabilitation as well as care if limited mobility remains, which is the case for 5 out of the 16 million people annually experiencing a first stroke around the globe [4].

Early post stroke, limited mobility can be caused by impaired trunk control [5]. Impaired trunk control is defined as the inability of the trunk muscles to maintain the body in an upright position, adjust weight-shift, or perform movements of the trunk, and encompasses an altered trunk position sense, poor trunk muscle strength, and balance difficulties [5, 6]. This inability to maintain the body in an upright position is also associated with impairments of functional outcomes such as balance, gait, arm, and hand function [6].

The initial severity of disability and extent of improvement observed within the first weeks post stroke were found to be substantial indicators of the functional outcome at six months [7–9]. It is also known that treatment intensity is important and neuroplasticity is greater early after stroke [10]. Additionally, the majority of motor recovery occurs during the first ten weeks after stroke. [7]. Therefore, early rehabilitation after stroke, especially of the trunk, is important. Because the impairment is higher early after stroke, selective trunk control exercises while sitting are suited best for trunk training during this rehabilitation stage. Trunk exercises, both on a stable or a mobile surface, have been shown to have a beneficial effect on trunk control, standing balance, mobility, dynamic sitting balance, and gait after stroke [11, 12]. This remains true even three months after cessation of regular training [11]. When the trunk control of people after stroke improves faster, gait and balance training can be started earlier and possibly lead to better recovery [13].

Particularly regarding standing and walking, selective trunk control, for example measured by the trunk impairment scale (TIS), is a predictor of the total functional outcome of rehabilitation [8, 9, 14]. Pelvic and lumbar control are prerequisites to proper movement control of the extremities which is needed in order to regain standing and walking abilities, or to adequately perform reaching [7, 15–17]. Another important predictor of motor and functional recovery after stroke is sitting balance which was identified in several studies [14, 18–20]. Training of sitting balance (while reaching beyond arm’s length) yields a positive effect on gait and mobility related functions and abilities [21]. Because lateral sitting balance appears to be more affected by stroke than anteroposterior sitting balance, possibly due to the lack of foot support and the consequent greater dependence on trunk muscles during lateral shifting, training of lateral sitting balance is needed more urgently during rehabilitation [22]. Sitting balance could be improved with trunk control training [23].

Currently, training tools, which are designed for selective trunk control training and can be used early after stroke or for severely impaired stroke survivors, are scarce. A novel assistive therapy chair, incorporating a mobile seat, allows trunk training in a seated position early in the rehabilitation process in a safe environment, and at a potentially higher training intensity [24]. Karatas et al. (2004) found a positive relationship between trunk muscle strength and the Berg Balance Scale at discharge, which shows the importance of trunk muscle strength training during rehabilitation [25].

The specific effects of selective trunk exercises using a mobile or a stable seat on the musculoskeletal system, specifically muscle activity of the trunk musculature, are currently unknown. Therefore, the aim of this exploratory study was two-folded. Primarily, the aim was to investigate whether the surface electromyographic activity (sEMG) of three trunk muscles during the performance of two selective lateral trunk control exercises is different on the mobile seat compared to the stable seat, in people after stroke (PAT) and in healthy participants (CON). The secondary aim was to find which of the factors seat condition, group, investigated side, or exercise type could predict trunk muscle activity.

## Materials and methods

### Participants

CON were recruited via mail from the university campus and wider public and PAT were recruited from the inpatient population of a rehabilitation center in Switzerland, both between September 2020 and March 2021. All participants were screened by verbal interview prior to enrolment based on preselected inclusion criteria (Table 1).

**Table 1:** Inclusion criteria.

Healthy participants	People after stroke
Age ≥ 18 years	Age ≥ 18 years
BMI 18–28	BMI 18–28
No acute or chronic musculoskeletal, neurological or cardiopulmonary diseases	No acute or chronic musculoskeletal, neurological or cardiopulmonary diseases
No scoliosis	No scoliosis
No pregnancy	No pregnancy
Being able to understand verbal and written instructions in German	Being able to understand verbal and written instructions in German
	Being able to perform at least 2h of rehabilitation training a day
	TIS score between 2–19 points

BMI = body mass index, TIS = trunk impairment scale

There were a total number of 28 participants of which 17 were male and 11 were female. Twenty-one participants (13 CON, 8 PAT) had a less affected/dominant right arm, and seven participants (2 CON, 5 PAT) had a less affected/dominant left arm. Other participant characteristics are displayed in Table 2.

**Table 2:** Means and standard deviations of participant characteristics.

Characteristic [unit]	N	Mean	SD
BMI [kg*m^-2^]	28	22.81	2.68
BMI people after stroke [kg*m^-2^]	13	22.57	3.55
BMI healthy participants [kg*m^-2^]	15	23.02	1.53
Age all participants [years]	27	47.85	22.56
Age people after stroke [years]	12	70.33	14.05
Age healthy participants [years]	15	29.87	5.34
TIS people after stroke [points]	12	12.67	3.84
Static sitting [points]	12	5.42	0.86
Dynamic sitting [points]	12	5.25	2.20
Coordination [points]	12	2.00	1.53

N = number of measurements, BMI = body mass index; TIS = trunk impairment scale; SD = standard deviation

Missing data of participant’s characteristics (age PAT, n = 1 and TIS PAT n = 1) resulted from missing data entry. Exhaustion (PAT on mobile seat, n = 1) and a misplaced electrode (Obliquus externus (OE) CON, n = 1) also led to missing data.

### Ethical considerations

This research complied with the tenets of the Declaration of Helsinki. The study was juristically verified by the medical Ethics Committee of Canton Zurich (Req-2020-00569). All participants provided their written informed consent.

### Apparatus

The movable seat is described in detail in separate publications and is therefore described only briefly here [24, 26]. The seat was designed for the rehabilitation of PAT to support the following therapy goals of the International Classification of Health Intervention (ICHI)—Interventions on Body Systems and Functions– 10 Interventions on the Musculoskeletal System–Movement (ICHI 1-10-MV) domain [27]:

- MVD.PG.ZZ Assisting and leading exercise for control of voluntary movement functions
- MVF.PG.ZZ Assisting and leading exercise for involuntary movement
- MVD.PH.ZZ Training motor control.

The movable seat possesses either one or two degrees of freedom i.e. in medio-lateral and antero-posterior direction or a combination thereof. The seat is mounted on a U-shaped rail and, thus, rotates around a virtual axis located at the spine, allowing the upper torso to be maintained in an upright, calm position during lateral and flexural motion of the lower spine and pelvis. The ergonomic design has been previously explained in detail [28, 29]. All necessary safety requirements (e.g. pinching of body parts, securing the patient) were implemented according to current technical standards [30–32]. The seat can be locked in place to provide a stable seat or unlocked for mobile sitting. The latter allows the person to move the seat freely or keep it stable through active control.

### Electromyography

Bilateral sEMG of M. multifidi (MF), M. erector spinae (ES), and M. obliquus externus were recorded using a myon sEMG system (myon AG Baar Switzerland; Type 142 RFTD-A01, D02-RFTD), at a sampling rate of 1200Hz and 12-bit resolution. To prepare the participants for sEMG measurements, the skin was first shaved and cleaned. Bipolar electrodes (Blue Sensor®, Ambu, Denmark, Type P-00- S, interelectrode distance: 20 148 mm) were then attached to the skin according to the recommendations of the sEMG of the Non-Invasive Assessment of Muscle Project (SENIAM) and Ng and colleagues [33, 34]. In both groups the same hardware was used, while the software for data collection was Vicon Nexus (Vicon, Oxford. UK, Version 2.11) in CON at the movement laboratory and proEMG (myon AG Baar Switzerland; Type 142 RFTD-A01, D02-RFTD) in PAT at the rehabilitation centre. Post processing was done with MATLAB software (MathWorks Inc., Natick, MA, USA, Version R2019A). The raw sEMG signals were high-pass, respectively low-pass filtered (Butterworth 2nd order, cut off frequency 30Hz respectively 500Hz). Subsequently, the root mean square (RMS), with a moving RMS window of 120 frames, was calculated and the maximal sEMG activity was then averaged from the five test repetitions in order to remove the power of potential intra-individual variability [35]. For PAT with possible muscle weakness, maximal voluntary contractions are not recommended to be performed [36, 37]. Therefore, maximal sEMG activity was calculated for each trial and then standardized to the maximal muscle activity of static sitting on the stable seat (STAT). The percentage of maximal muscle activity relative to STAT (%STAT) was calculated as outcome variable. This was done to assess whether any given muscle was more or less active in comparison to static sitting, depending on the group, exercise, body side, or seat condition.

### Trunk control exercises

**Fig 1:**
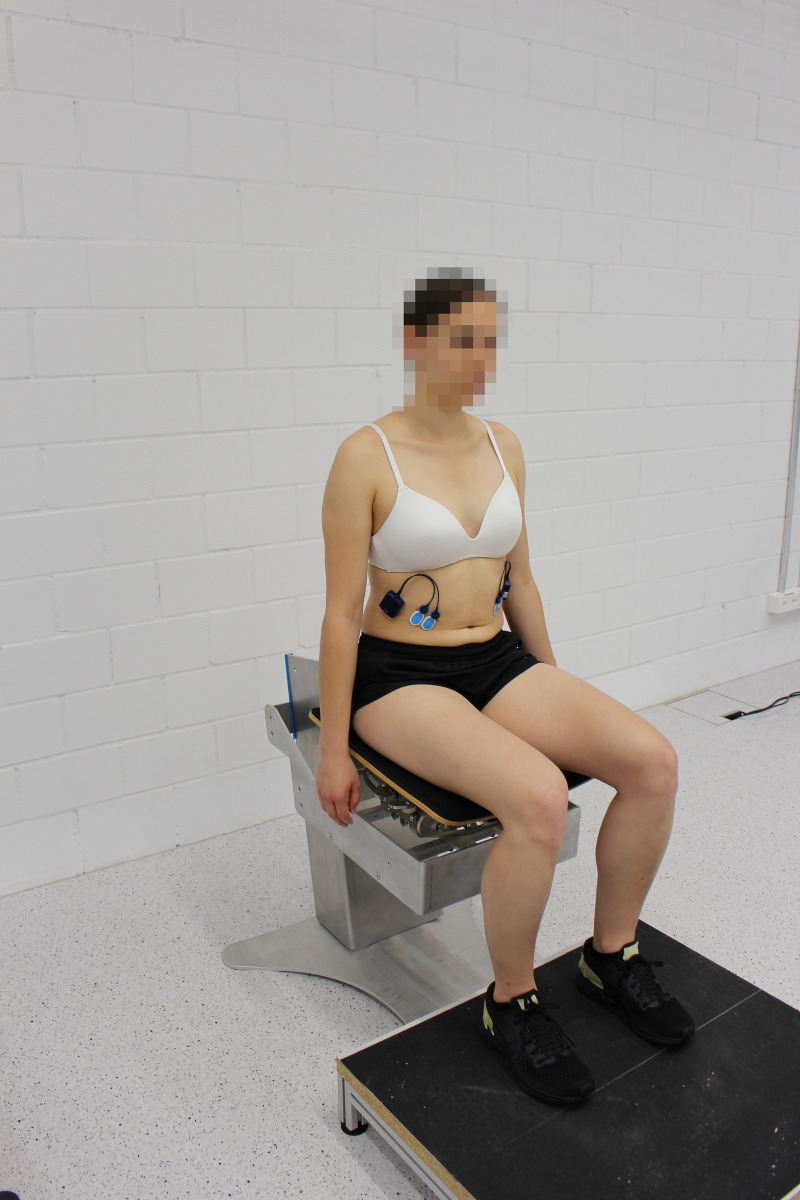
Participant sitting in the static sitting position on the stable seat.

**Fig 2:**
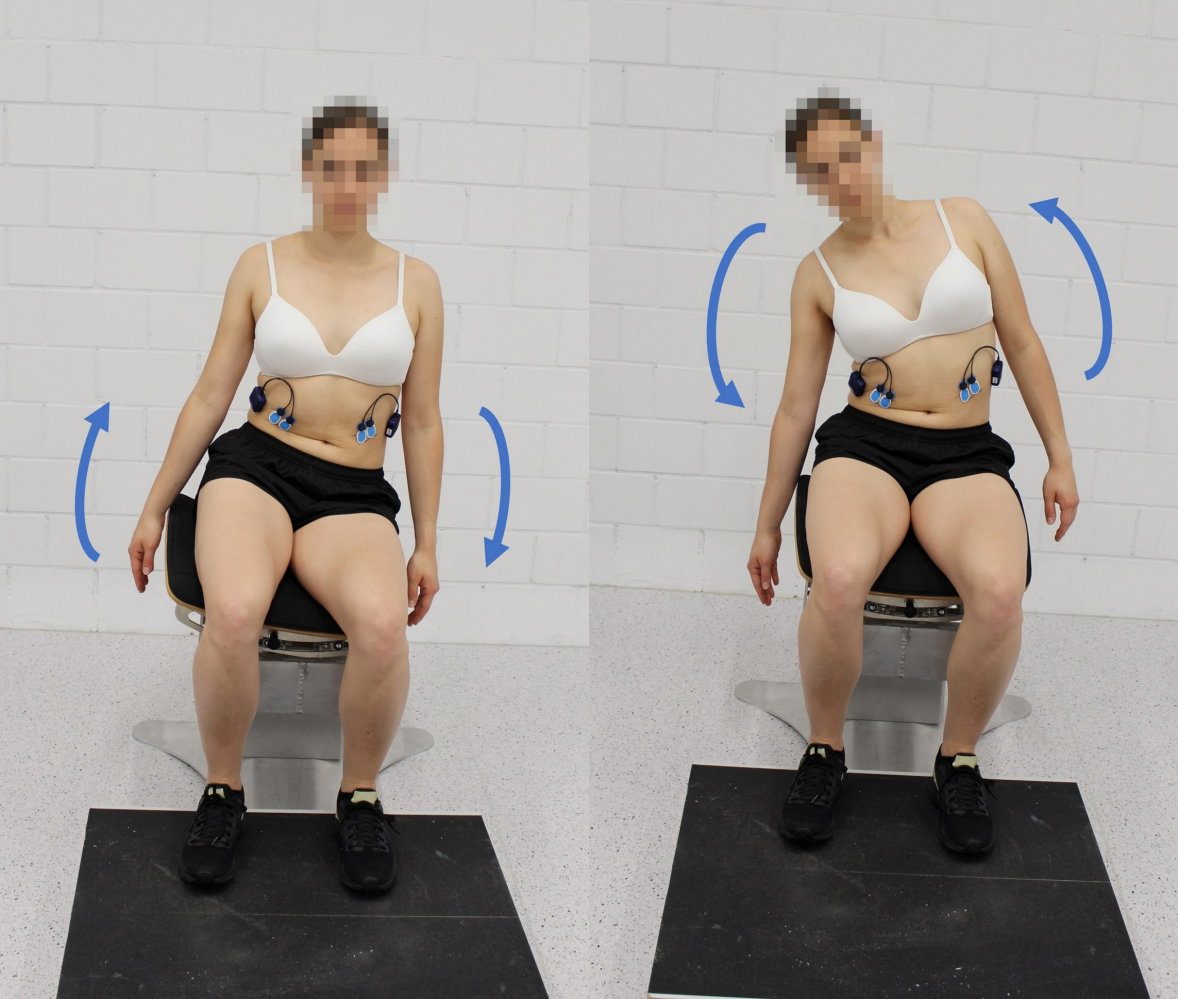
Trunk control exercises with movement initiated by the pelvis (left) and movement initiated by the trunk (right) on the mobile seat.

During the one measurement session, participants received standardized oral instructions from one of the examiners and were able to practice the exercises prior to the tests. Each participant performed first STAT (Fig 1) and then two trunk control exercises, both on the stable and on the mobile seat with five repetitions. In the first exercise, participants were asked to initiate an isolated lateral flexion (LF) of their pelvis, and in the second to initiate an isolated LF of their trunk. If initial performance was poor, the instructions were repeated, and the participant received tactile feedback. During the “lateral trunk flexion” the participants sat upright on the seat, with their hip and knee angles flexed between 80° - 90°, their arms resting at their sides, and their feet placed on a footstool at hip width (Fig 2).

During the pelvis-initiated LF, participants were instructed to tilt the seat as far as possible to both sides while maintaining the upper body upright and as steady as possible. For the thorax-initiated LF, participants were instructed to reach as far as possible from the side of the chair to the floor, while keeping the seat surface as steady as possible and their full feet on the ground. In this way, the thorax was flexed, first to the right and then to the left, while the lumbar spine and pelvis were maintained as steady as possible. The order of the exercises was fixed for each participant, firstly on the stable and then on the mobile seat.

### Statistical analysis

A 3-way within-subject linear mixed model was fitted to the data of each muscle separately with %STAT as the outcome of interest *Y*_*i*_. In a first model all main effects were included, with *β*_0_ representing the intercept, *β*_*k*_ the effect of the k-covariate and *ε*_*i*_ the independent and normal distributed errors *ε*_*i*_ ~ *N*(0, *σ*^2^). The covariates consisted of the between subject covariate: Group, the within-subject covariates: Side, Exercise, and Seat Condition, and the 2-way, 3-way, and 4-way fixed interaction effects.


$$\begin{array}{l} Y_{i}=\beta_{0}+\beta_{1}Group_{i}+\beta_{2}Side_{j}+\beta_{3}Seat_{Condition_{j}}+\beta_{4}Exercise_{j}\\ \;+\beta_{5}Group_{i}xSide_{j}+\beta_{6}Group_{i}xSeat\_Condition_{j}\\ \;+\beta_{7}Group_{j}xExercise_{j}+\beta_{8}Side_{j}xSeat\_Condition_{j}\\ \;+\beta_{9}Side_{j}xExercise_{j}+\beta_{10}Exercise_{j}xSeat\_Condition_{j}\\ \;+\beta_{11}Group_{i}xSide_{j}xSeat\_Condition_{j}\\ \;+\beta_{12}Group_{i}xSide_{j}xExercise_{j}\\ \;+\beta_{13}Group_{i}xExercise_{j}xSeat\_Condition_{j}\\ \;+\beta_{14}Exercise_{j}xSide_{j}xSeat\_Condition_{j}\\ \;+\beta_{15}Exercise_{j}xSide_{j}xSeat\_Condition_{j}xGroup_{i}+\varepsilon_{i} \end{array}$$

Each covariate had two levels, which were CON and PAT for group, less affected/dominant and affected/non-dominant for side, mobile and stable for seat condition, and pelvis- and thorax-initiated for exercise. The less affected side of PAT was equated to the dominant side of CON, whereas the affected side of PAT was equated to the non-dominant side of CON. A stepwise model selection procedure, with backwards optimization through exclusion of non-significant covariates and the Akaike-Information-criterion was used to determine the final model. The aim of this procedure was to choose a parsimonious model to prevent overfitting of the data and ensure that the model is optimized for prediction, that is, for future data.

Descriptive statistics and statistical analysis were calculated with R Version 4.10 [38] and the packages lme4 [39], lmerTest [40], psych [41], emmeans [v1.6.2–1; 42], and ggplot2 [43]. Likelihood ratio tests revealed that 2-way, 3-way, and 4-way interactions could not be removed for MF, but for ES and OE the 4-way interaction could be removed. For ES, the factor side could be removed as well, resulting in a better model fit. In OE, a better model fit resulted when interactions with side were removed. The outcome variable was logarithmically transformed due to the distribution of the residuals. Significance level was set to p < 0.05.

## Results

Descriptive data of each subgroup and each muscle can be found in the supplementary material. Additionally, predictive values and interaction plots are also available in the supplementary material. The results which are presented in this section all correspond to the predicted %STAT.

### M. multifidi

There was a significant 4-way interaction effect for all factors (seat condition, group, side, and exercise), *F*(1, 28.55) = 11.67, p < .002. Additionally, a 3-way interaction effect for seat condition, group, and exercise was found, *F*(1, 25.48) = 12.47, p < .002.

**Fig 3:**
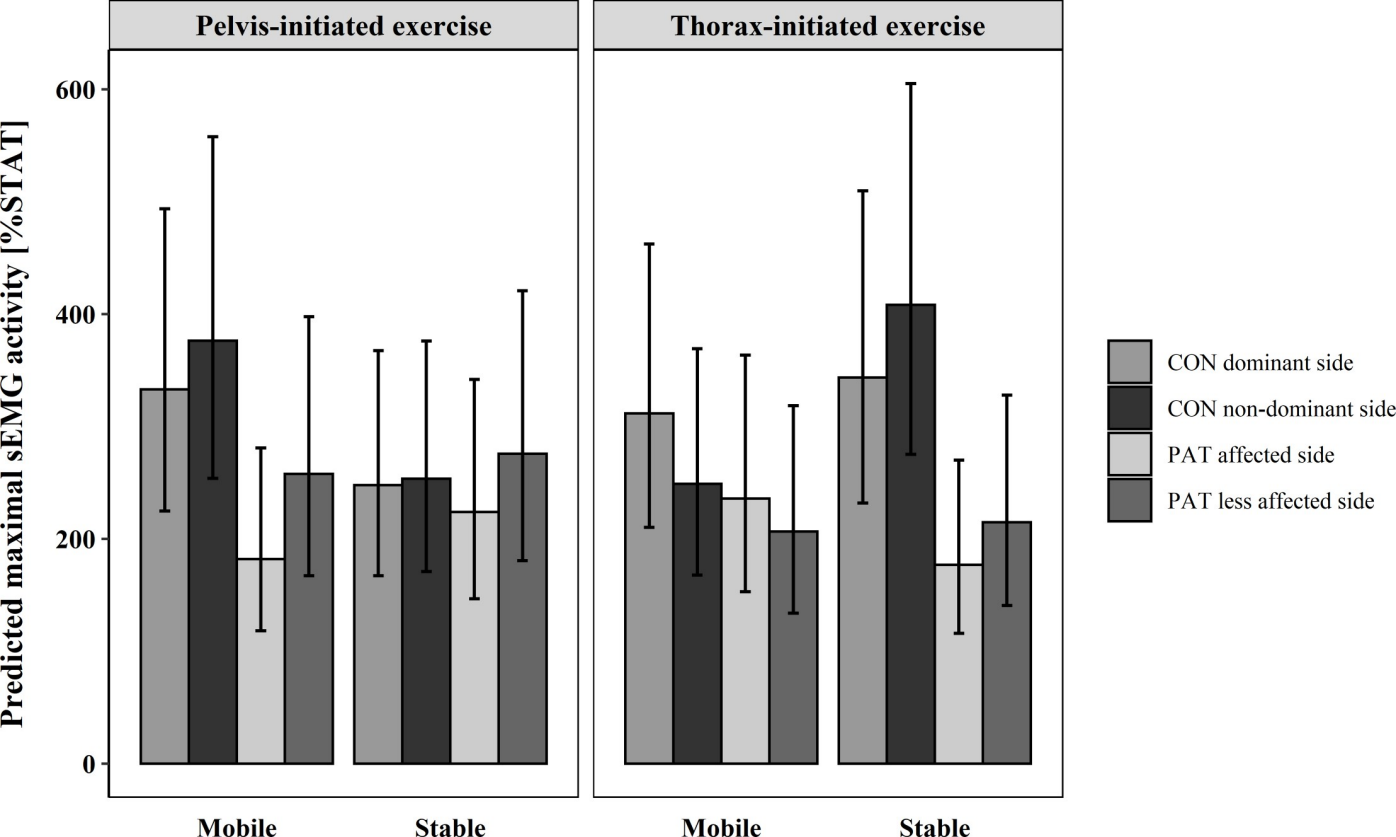
Predicted maximal sEMG activity and confidence level of each subgroup in M. multifidi. CON = healthy participants, PAT = people after stroke, %STAT = percentage of maximal muscle activity relative to static sitting on the stable seat.

As can be seen in Fig 3, the predicted %STAT in pelvis-initiated LF on the mobile seat was higher for CON and lower for PAT, compared to the stable seat. Thorax-initiated LF showed opposite results than pelvis-initiated LF, with lower %STAT on the mobile seat. The exception was the affected side of PAT where %STAT was higher on the mobile seat. Small differences between sides were present.

On the mobile seat peak muscle activity for CON during thorax-initiated LF was 2.5–3.1 times higher than in static sitting, respectively 2.1–2.4 times higher for PAT, whereas on the stable seat it was 3.4–4.1 times higher for CON and 1.8–2.1 times higher for PAT. CON showed 3.3–3.8 times higher peak muscle activity during pelvis-initiated LF than during static sitting on the mobile seat, while on the stable seat peak muscle activity was only 2.5 times higher. With 1.8–2.6 times higher activity on the mobile seat and 2.2–2.5 times higher activity on the stable seat, PAT showed lower variation than CON.

### M. erector spinae

A statistically significant 3-way interaction effect for group, exercise, and seat condition was found for ES, *F*(1, 25.66) = 6.95, p = .014, as well as a 2-way interaction effect for exercise and group *F*(1, 26.24) = 7.52, p = .011.

**Fig 4:**
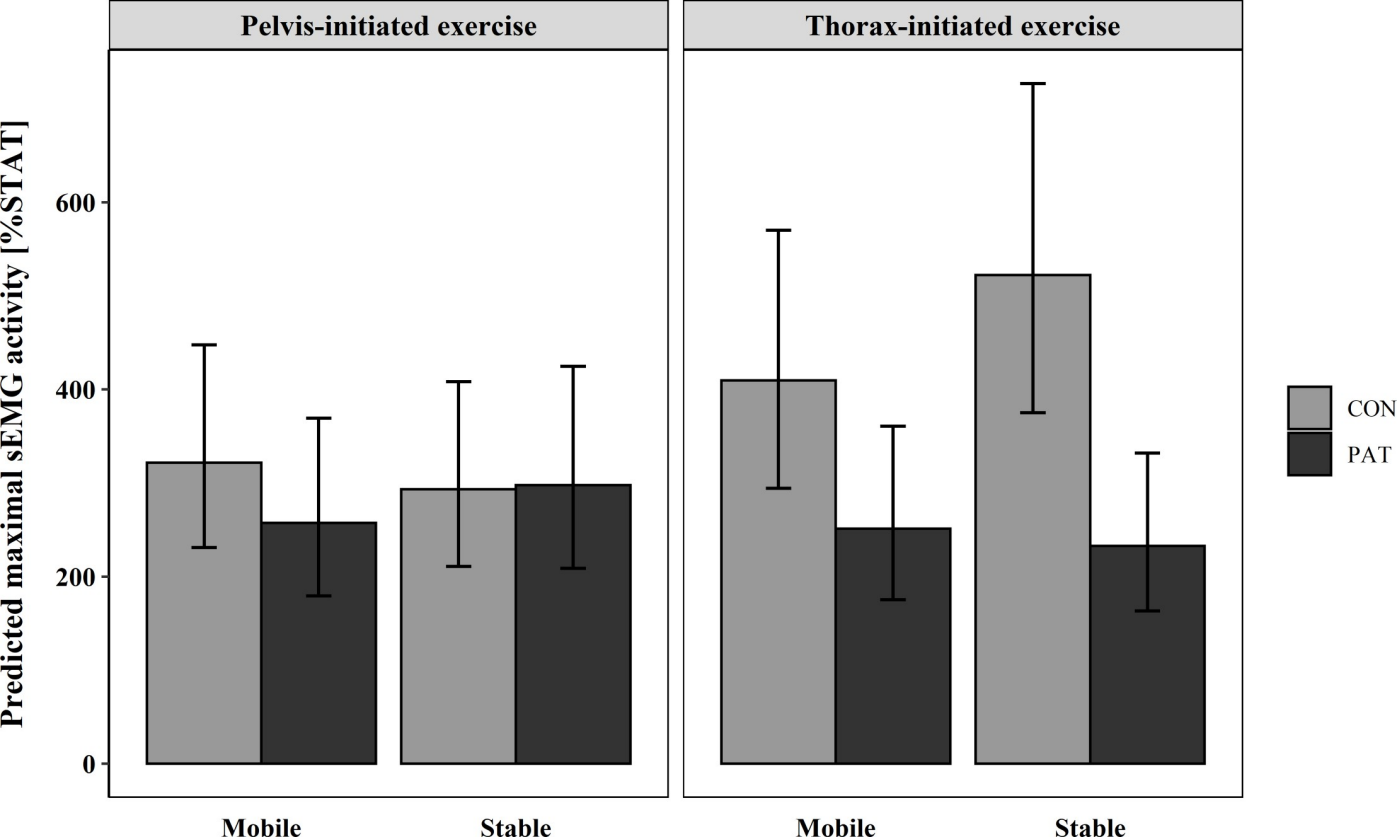
Predicted maximal sEMG activity and confidence level of each subgroup in M. erector spinae. CON = healthy participants, PAT = people after stroke, %STAT = percentage of maximal muscle activity relative to static sitting on the stable seat.

Whereas the %STAT of ES on the mobile seat was higher for the pelvis-initiated LF in CON and for the thorax-initiated LF in PAT, higher %STAT on the stable seat was seen for the thorax-initiated LF in CON and for the pelvis-initiated LF in PAT (Fig 4). Additionally, CON had a higher %STAT than PAT, except for the pelvis-initiated exercise on the stable seat where the difference was very small. In general, the %STAT was higher for the thorax-initiated exercise in CON and for the pelvis-initiated exercise in PAT. Compared to static sitting, PAT had 2.3–3 times higher peak muscle activity, whereas CON had 2.9–5.2 times higher peak muscle activity, depending on the exercise and seat condition.

### M. obliquus externus

OE showed a significant main effect for both group, *F(*1, 25.67) = 33.81, p < .001, and exercise *F(*1, 24.59) = 38.65, p < .001. Additionally, a significant interaction effect was found for the factors group and exercise, *F(*1, 24.59) = 31.39, p < .001, and seat condition, group, and exercise, *F(*1,24.11) = 6.62, p = 0.017.

**Fig 5:**
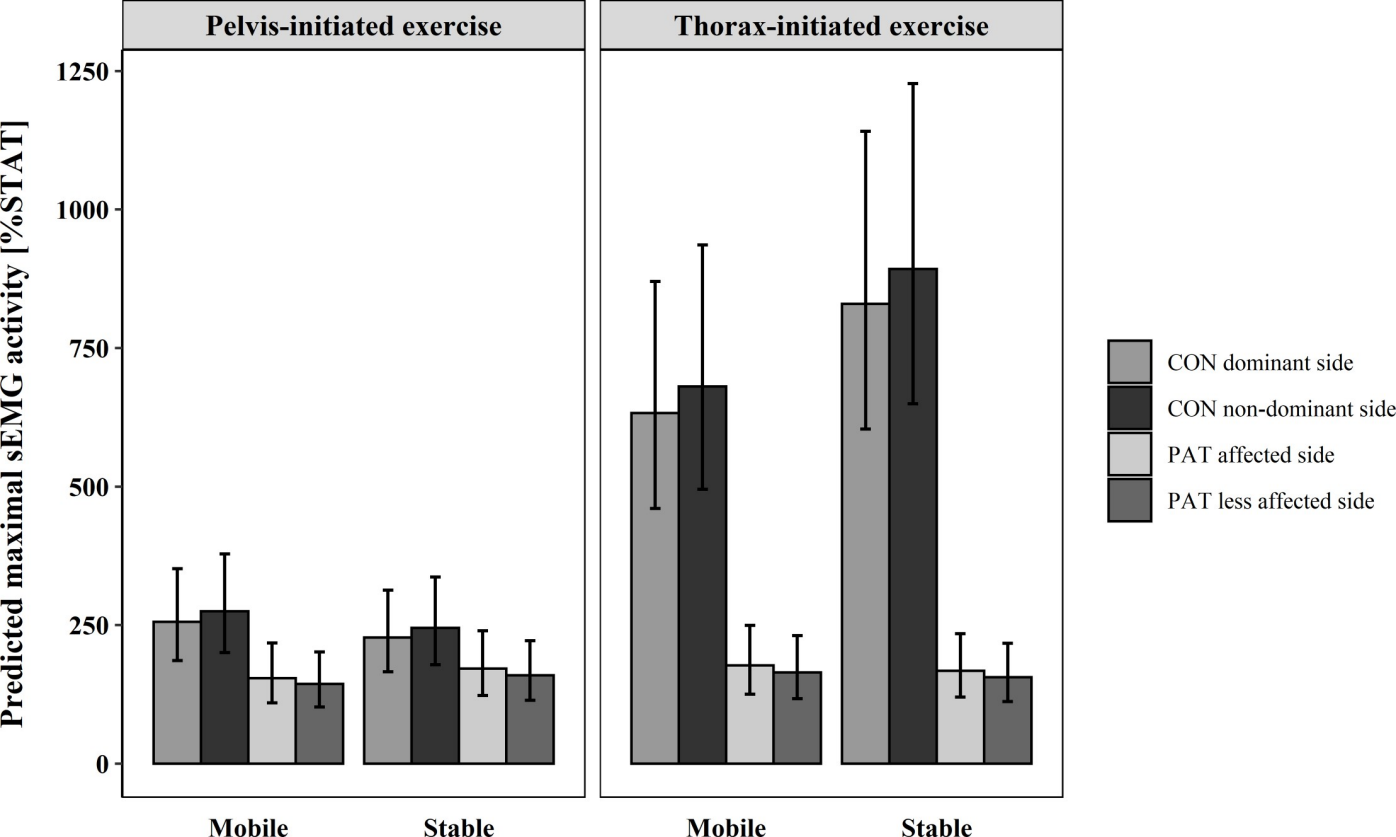
Predicted maximal sEMG activity and confidence level of each subgroup in M. obliquus externus. CON = healthy participants, PAT = people after stroke, %STAT = percentage of maximal muscle activity relative to static sitting on the stable seat.

In both seat conditions and exercises, PAT showed a similar %STAT (Fig 5). In contrast, CON had a higher %STAT on the stable seat for the thorax-initiated LF, but not for the pelvis-initiated LF. A difference between groups for the thorax-initiated LF existed, with much higher %STAT of CON. Otherwise differences were much smaller. The %STAT of CON during the thorax-initiated LF was much higher than the %STAT of PAT. Side differences were very small on the stable seat, but on the mobile seat CON showed higher %STAT on the dominant side.

Whereas during the thorax-initiated LF of CON, peak muscle activity on the stable seat was 6.3–6.8 times the size of peak muscle activity during static sitting, respectively 8.3–8.9 times the size on the mobile seat, during the pelvis-initiated LF the peak muscle activity was only 2.3–2.8 times as high as the peak activity of static sitting. For PAT, peak muscle activity is 1.4–1.8 times higher on the mobile seat and 1.6–1.7 times higher on the stable seat.

## Discussion

To the author’s knowledge, this is the first study to investigate bilateral %STAT of trunk muscles comparing a stable and mobile seat during seated trunk control exercises, namely thorax- and pelvis-initiated LF, in both CON and PAT. Our research has shown that %STAT in PAT is higher on the mobile seat during the thorax-initiated LF and lower during the pelvis-initiated LF. In contrast, higher %STAT on the mobile seat was present in CON during the pelvis-initiated LF and lower during the thorax-initiated LF. But our results have also shown that during seated trunk control exercises, seat condition, exercise, and group affect the %STAT of trunk muscles. However, due to the 4-way interaction which was only present in MF, the investigated side does only interact with other factors in these muscles. 3-way interactions between different investigated factors were found in all three muscles. For this reason, it cannot be stated in which seat condition, side, group, or exercise %STAT is the highest, since it is dependent on the other factors.

Performing the exercise on the mobile seat, compared to the stable seat, led to higher %STAT during pelvis-initiated LF in CON, while for PAT this was the case when performing thorax-initiated LF. %STAT could either be higher on the mobile seat due to a possibly larger range of motion or due to more stabilization effort to keep the body in a stable position. Since there were differences depending on group and exercise, it is assumed that the larger range of motion only played a small role and primarily the stabilization effort of the muscles resulted in higher %STAT. It seems like CON use MF, ES, and OE to stabilize the upper part of the trunk on the mobile seat while moving their pelvis laterally, and PAT use these muscles to stabilize their hip and lower trunk when moving the thorax laterally. Existing literature suggests that the cross-sectional area of back muscles (MF) and the muscle activity of trunk muscles (upper and lower rectus abdominis) after training on a mobile surface like a Swiss Ball or plinth is increased compared to training on a stable surface [44, 45]. In a systematic review, Van Criekinge et al. (2019) reported that only the muscle activity of internal oblique muscles improved significantly after trunk training. Although not significantly, ES, OE, and rectus abdominis muscle activity still increased after trunk training, independent of the sitting surface. They concluded that, regarding the increase of muscle thickness and muscle activity, training on a mobile surface was more efficient than training on a stable surface [46]. Even though for each group only one exercise led to higher %STAT on the mobile seat, prior research suggests that training for a longer period on a mobile seat could be more efficient [44–46].

While during the pelvis-initiated LF, %STAT was around the same size in all muscles, during the thorax-initiated LF, %STAT differed between the muscles. In CON, with values between 632.86 and 892.63%STAT, the predicted %STAT was generally much higher in OE compared to MF and ES with values only between 248.92 and 522.33%STAT. Previous research has found that obliques act as prime movers during lateral bending [47], which could explain the higher %STAT of OE muscles compared to other muscles during thorax-initiated LF in CON. PAT showed similar %STAT in both exercises for MF and ES, only for OE %STAT was somewhat smaller than the two back muscles. This could either mean that PAT compensated with other muscles, that were not measured, or that they had a smaller range of movement either due to limited muscle strength or due to limited trunk control. Nonetheless, in all conditions and muscles the predicted %STAT was at least 1.4 times higher than peak muscle activity during static sitting, which suggests that the trunk musculature is engaged during lateral flexion exercises and could therefore result in a training effect.

Differences of %STAT between the affected and the less affected side exist for PAT, except for ES for which the covariate side could be removed. PAT have small coherence of antagonistic muscle pairs which could explain these findings [48]. Our findings that for ES the side difference was not significant, does not concur with the results of the study of Winzeler-Merçay and Mudie (2002). They compared muscle activity during sitting exercises in PAT and CON, which led to the conclusion that, for PAT, ES activation is higher on the affected side. [37]. In our study, the less affected side only showed higher %STAT in MF, which was not measured in their study. Cai et al. (2019) reported that the less affected side of OE and ES showed less average muscle activity than the affected side in PAT [49], which is partially in line with our results since ES did not show any side differences. Possible reasons for this difference are that the performed exercise in our study was LF initiated from the thorax and from the pelvis, while in the study of Cai et al. (2019) side-to-side, up-and-down, and back-and-forth reaching exercises were performed and in the study of Winzeler-Merçay and Mudie (2002) sitting exercises were performed [37, 49]. Additionally, different outcomes of sEMG measurements were used.

In our CON group, significant side differences of %STAT were only present in MF and OE. While in OE %STAT was higher on the non-dominant side, in MF this was dependent on the exercise and seat. Both exercises were symmetrical which is why side differences in CON were surprising. Existing literature investigating trunk muscle activity or cross-sectional area of similar tasks showed side differences as well. Hides et al. (2008) found 5.2% asymmetry in the cross-sectional area of MF at the L2 vertebra of CON, while Farahpour et al. (2015) found a significant difference in agonistic and antagonistic muscle activity of ES in young and healthy controls during lateral bending while standing [50, 51]. Similar results were found by Kuriyama and Ito (2005) who stated that contralateral muscle activity, but not ipsilateral muscle activity, was present when bending laterally in healthy controls [52]. Both exercises were performed to the right and left side and are therefore symmetrical, which suggests that both sides were contralateral to the movement in one part of the exercise. Additionally, Pereira et al. (2011) found no difference in muscle activation onset between ipsi- and contralateral sides for symmetrical exercises [53] and therefore, the timing of both sides should be similar. However, Peach et al. (1998) state that peaking of muscle activity happens at different timepoints. While ipsilateral muscles peak at the change of direction, contralateral muscles peak during the rising movement [47]. In CON it can be assumed that muscle strength is similar in both sides and consequently %STAT of both sides occurred at the same point of the movement, either at the ipsilateral change of direction or the contralateral rising movement.

Exercise showed interactions with other factors in all three muscles. Therefore, a general statement regarding which exercise leads to lower %STAT, cannot be made. Contrast analysis for exercise differences showed that ES and OE behaved similarly, which is surprising due to the fact that ES is a lateral trunk extensor and OE is a trunk flexor and hence their function is different [53]. CON have higher %STAT during thorax-initiated LF while PAT have slightly higher %STAT during pelvis-initiated LF. For MF and OE (only of PAT), the seat condition plays an additional role. Thorax-initiated LF leads to higher %STAT on the stable seat in CON while in PAT higher %STAT is reached on the mobile seat. Higher %STAT of pelvis-initiated LF was seen in the corresponding conditions. The fact that, even though both exercises represent a trunk control exercise, the moving body parts are contrary to each other could explain the contrasting result. While during the thorax-initiated LF, the lumbar spine and pelvis were held as stable as possible, during the pelvis-initiated LF the thoracic and cervical spine were kept as stable as possible. When the sitting position is changed from slouched to upright through a lumbopelvic initiated pattern, the load (and possibly also the muscle activity) is smaller due to the proximity to neutral posture [54]. This fact could indicate that in CON, the range of motion during the pelvis-initiated LF is lower and that muscle activity is therefore lower as well. It is possible that the thorax-initiated LF on the mobile seat led to sensory inputs which were difficult to process for PAT and hence muscles were activated more even though it was possibly not necessary. Another possible explanation would be that pelvis-initiated LF was perceived as difficult by PAT due to sensory deficits after stroke and therefore, movement was kept to a minimum which resulted in lower %STAT.

The participant characteristics of the two groups investigated in this study were very different. It is likely that the observed group difference resulted due to the contribution of several factors as age, lifestyle, health condition or gender. Mean body mass index values of both groups were similar. CON were young, while PAT were older and, due to their condition, probably more sedentary. A sedentary lifestyle increases the risk of more subcutaneous fat which in turn, increases the risk of measurement error of sEMG [55]. But because mean body mass index values were similar in both groups, this difference is expected to have a minimal impact. Furthermore, the mean score in the dynamic sitting subscale of the trunk impairment scale was only 5.25 from a maximum of 10 points. Since dynamic sitting exercises of the TIS are similar to our trunk control exercises, it can be assumed that PAT were limited in their trunk control and probably used compensation strategies which are shown through diverging muscle activity of the three investigated muscles. Also, trunk recovery is different depending on the side of hemiparesis [56]. Different stages of recovery in the PAT group could therefore explain a high variability of %STAT. With increasing age, muscles get activated earlier and there is more co-contraction. Additionally, the peak displacement for lateral bending is lower in older people [57]. Lower peak displacement suggests a lower muscle activity since the deviation from the stable, upright sitting axis is smaller. Thus, the muscular effort to return to this stable body axis is smaller which could explain differences between groups. Differences between males and females in sitting and standing posture have been reported [58], but the effect of an imbalance in the number of male and female participants is expected to be minimal since each participant’s muscle activity was referenced to their own static sitting muscle activity.

Several limitations were present in the current study. Muscle activity was not normalized to each participants’ individual maximal muscle strength, but to the %STAT without specific instructions on how much the muscles should be tensed, which might lead to a high variability of the data. Maximal voluntary contraction was not measured because of PAT’s physical conditions and because physical contact was reduced to a minimum due to Covid-19. From literature, it is known that static sitting posture of PAT is less stable than static sitting posture of CON [59]. Therefore, higher variability of STAT could counteract variability of %STAT in PAT and in CON stable STAT and %STAT is assumed. Our sample size was small with only 15 CON and 13 PAT, which leads to a limited generalizability of results. The COVID-19 pandemic restricted the recruitment of participants, both in terms of the sample size and matching between the groups, for example in terms of age. Right-handed participants represented the majority and left-handed participants were underrepresented in the CON group. In PAT, the affected side was almost balanced between right and left. Furthermore, the dominant side of CON was equated with the less affected side of PAT and the non-dominant side of CON with the affected side of PAT, which might not be true in terms of muscle strength or muscle activity. In other studies, comparing sides of PAT and CON, the same approach was chosen [37]. Muscular activity is not always equivalent to muscle strength, especially in PAT, and it is therefore not known whether the mobile surface leads to greater trunk muscle strength. However, an almost linear relationship between sEMG amplitude and muscle force exists [60]. For this reason, it can be assumed that higher peak sEMG values lead to higher muscle force generation and that trunk muscles are more engaged when peak sEMG activity is high which in turn increases the possible training effect. Other limitations were usual limits of sEMG procedure, for example the influence of thermal conditions. The measurement protocol was designed in order to achieve measurement sessions which are as similar as possible.

In general, high standard deviations in our data suggest great variability between individuals and, therefore, the results must be interpreted cautiously. Further work with PAT and CON groups with a higher number of participants could clarify whether our results are generalizable. Similarly, studies with more than one measurement session could indicate whether there is a different training effect for each seat on trunk muscle activity as well as muscle strength.

## Conclusion

Trunk control exercises on a mobile sitting surface could be an effective and safe training method for both PAT and for CON. Depending on the group and exercise, the mobile or the stable sitting surface leads to higher %STAT. When a high muscular activity and therefore high muscle engagement is intended on the mobile surface, PAT are recommended to perform the thorax-initiated LF while CON are recommended to perform the pelvis-initiated LF. If, in contrast, a low muscle activity and therefore low muscle engagement on the mobile seat is intended, it is recommended that CON perform the thorax-initiated LF and PAT the pelvis-initiated LF. Further studies are needed to clarify which factors contribute to an effective training program including multiple sessions on the different sitting surfaces and whether training on one surface is more effective than on the other.

## Supporting information

S1 File Supplementary material–interaction plots. (PDF)

S2 File Supplementary material–predicted and descriptive sEMG data. (DOCX)

S3 File Minimal dataset. (XLSX)

## References

[1] Feigin VL, Stark BA, Johnson CO, Roth GA, Bisignano C, Abady GG, et al. Global, regional, and national burden of stroke and its risk factors, 1990–2019: a systematic analysis for the Global Burden of Disease Study 2019. Lancet Neurol. 2021 Oct 1;20(10):795–820. doi: 10.1016/S1474-4422(21)00252-0 34487721 PMC8443449

[2] Feigin VL, Krishnamurthi RV, Parmar P, Norrving B, Mensah GA, Bennett DA, et al. Update on the Global Burden of Ischemic and Hemorrhagic Stroke in 1990–2013: The GBD 2013 Study. Neuroepidemiology. 2015;45(3):161–76. doi: 10.1159/000441085 26505981 PMC4633282

[3] Miller EL, Murray L, Richards L, Zorowitz RD, Bakas T, Clark P, et al. Comprehensive overview of nursing and interdisciplinary rehabilitation care of the stroke patient: a scientific statement from the American Heart Association. Stroke. 2010 Oct;41(10):2402–48. doi: 10.1161/STR.0b013e3181e7512b 20813995

[4] Strong K, Mathers C, Bonita R. Preventing stroke: saving lives around the world. Lancet Neurol. 2007 Feb 1;6(2):182–7. doi: 10.1016/S1474-4422(07)70031-5 17239805

[5] Karthikbabu S, Chakrapani M, Ganeshan S, Rakshith KC, Nafeez S, Prem V. A review on assessment and treatment of the trunk in stroke: A need or luxury. Neural Regen Res. 2012 Sep 5;7(25):1974–7. doi: 10.3969/j.issn.1673-5374.2012.25.008 25624827 PMC4298892

[6] Davies PM. Right in the middle: selective trunk activity in the treatment of adult hemiplegia. Springer Science & Business Media; 1990.

[7] Kwakkel G, Kollen BJ. Predicting activities after stroke: what is clinically relevant? Int J Stroke. 2013;8(1):25–32. doi: 10.1111/j.1747-4949.2012.00967.x 23280266

[8] Kim TJ, Seo KM, Kim DK, Kang SH. The relationship between initial trunk performances and functional prognosis in patients with stroke. Ann Rehabil Med. 2015 Feb;39(1):66–73. doi: 10.5535/arm.2015.39.1.66 25750874 PMC4351497

[9] Verheyden G, Vereeck L, Truijen S, Troch M, Herregodts I, Lafosse C, et al. Trunk performance after stroke and the relationship with balance, gait and functional ability. Clin Rehabil. 2006;20(5):451–8. doi: 10.1191/0269215505cr955oa 16774097

[10] Verheyden G, Nieuwboer A, De Wit L, Thijs V, Dobbelaere J, Devos H, et al. Time Course of Trunk, Arm, Leg, and Functional Recovery After Ischemic Stroke. Neurorehabil Neural Repair. 2008 Mar 1;22(2):173–9. doi: 10.1177/1545968307305456 17876069

[11] Cabanas-Valdés R, Bagur-Calafat C, Girabent-Farrés M, Caballero-Gómez FM, du Port de Pontcharra-Serra H, German-Romero A, et al. Long-term follow-up of a randomized controlled trial on additional core stability exercises training for improving dynamic sitting balance and trunk control in stroke patients. Clin Rehabil. 2017 Nov 1;31(11):1492–9. doi: 10.1177/0269215517701804 28351168

[12] Haruyama K, Kawakami M, Otsuka T. Effect of Core Stability Training on Trunk Function, Standing Balance, and Mobility in Stroke Patients. Neurorehabil Neural Repair. 2017;31(3):240–9. doi: 10.1177/1545968316675431 27821673

[13] Saeys W, Vereeck L, Truijen S, Lafosse C, Wuyts FP, Heyning PV. Randomized controlled trial of truncal exercises early after stroke to improve balance and mobility. Neurorehabil Neural Repair. 2012 Apr;26(3):231–8. doi: 10.1177/1545968311416822 21844283

[14] Kwakkel G, Wagenaar RC, Kollen BJ, Lankhorst GJ. Predicting disability in stroke—a critical review of the literature. Age Ageing. 1996;25(6):479–89. doi: 10.1093/ageing/25.6.479 9003886

[15] Verheyden G, Nieuwboer A, De Wit L, Feys H, Schuback B, Baert I, et al. Trunk performance after stroke: an eye catching predictor of functional outcome. J Neurol Neurosurg Psychiatry. 2007;78(7):694–8. doi: 10.1136/jnnp.2006.101642 17178824 PMC2117706

[16] Chuter VH, Janse de Jonge XAK. Proximal and distal contributions to lower extremity injury: A review of the literature. Gait Posture. 2012 May 1;36(1):7–15. doi: 10.1016/j.gaitpost.2012.02.001 22440758

[17] Kibler WB, Press J, Sciascia A. The Role of Core Stability in Athletic Function. Sports Med. 2006 Mar 1;36(3):189–98. doi: 10.2165/00007256-200636030-00001 16526831

[18] Wade DT, Skilbeck CE, Hewer RL. Predicting Barthel ADL score at 6 months after an acute stroke. Arch Phys Med Rehabil. 1983;64(1):24–8. 6849630

[19] Feigin L, Sharon B, Czaczkes B, Rosin AJ. Sitting equilibrium 2 weeks after a stroke can predict the walking ability after 6 months. Gerontology. 1996;42(6):348–53. doi: 10.1159/000213814 8930622

[20] Hsieh CL, Sheu CF, Hsueh IP, Wang CH. Trunk control as an early predictor of comprehensive activities of daily living function in stroke patients. Stroke. 2002;33(11):2626–30. doi: 10.1161/01.str.0000033930.05931.93 12411652

[21] Veerbeek JM, van Wegen E, van Peppen R, van der Wees PJ, Hendriks E, Rietberg M, et al. What is the evidence for physical therapy poststroke? A systematic review and meta-analysis. PloS One. 2014;9(2):e87987. doi: 10.1371/journal.pone.0087987 24505342 PMC3913786

[22] van Nes IJ, Nienhuis B, Latour H, Geurts AC. Posturographic assessment of sitting balance recovery in the subacute phase of stroke. Gait Posture. 2008 Oct;28(3):507–12. doi: 10.1016/j.gaitpost.2008.03.004 18424149

[23] Cabanas-Valdés R, Cuchi GU, Bagur-Calafat C. Trunk training exercises approaches for improving trunk performance and functional sitting balance in patients with stroke: A systematic review. NeuroRehabilitation. 2013;33(4):575–92. doi: 10.3233/NRE-130996 24018373

[24] Bauer CM, Nast I, Scheermesser M, Kuster RP, Textor D, Wenger M, et al. A novel assistive therapy chair to improve trunk control during neurorehabilitation: Perceptions of physical therapists and patients. Appl Ergon. 2021 Jul 1;94:103390. doi: 10.1016/j.apergo.2021.103390 33640840

[25] Karatas M, Cetin N, Bayramoglu M, Dilek A. Trunk muscle strength in relation to balance and functional disability in unihemispheric stroke patients. Am J Phys Med Rehabil. 2004 Feb;83(2):81–7. doi: 10.1097/01.PHM.0000107486.99756.C7 14758293

[26] Thijs L, Voets E, Wiskerke E, Nauwelaerts T, Arys Y, Haspeslagh H, et al. Technology-supported sitting balance therapy versus usual care in the chronic stage after stroke: a pilot randomized controlled trial. J NeuroEngineering Rehabil. 2021 Jul 28;18(1):120. doi: 10.1186/s12984-021-00910-7 34321042 PMC8316712

[27] Hart T, Dijkers MP, Whyte J, Turkstra LS, Zanca JM, Packel A, et al. A Theory-Driven System for the Specification of Rehabilitation Treatments. Arch Phys Med Rehabil. 2019 Jan;100(1):172–80. doi: 10.1016/j.apmr.2018.09.109 30267669

[28] Bauer CM, Rast FM, Bock C, Kuster RP, Baumgartner D. Determination of a sagittal plane axis of rotation for a dynamic office chair. Appl Ergon. 2018 Oct;72:107–12. doi: 10.1016/j.apergo.2018.05.008 29885721

[29] Kuster RP, Bauer CM, Oetiker S, Kool J. Physiological Motion Axis for the Seat of a Dynamic Office Chair. Hum Factors. 2016 Sep;58(6):886–98. doi: 10.1177/0018720816646508 27150530 PMC4971607

[30] IEC. IEC 80601-2-78:2019 [Internet]. 80601 [cited 2020 Nov 9]. Available from: https://www.iso.org/cms/render/live/en/sites/isoorg/contents/data/standard/06/84/68474.html

[31] ISO. ISO 14971:2007 [Internet]. 14971 [cited 2020 Nov 8]. Available from: https://www.iso.org/cms/render/live/en/sites/isoorg/contents/data/standard/03/81/38193.html

[32] ISO. ISO 24496:2017 [Internet]. 24496 [cited 2020 Nov 9]. Available from: https://www.iso.org/cms/render/live/en/sites/isoorg/contents/data/standard/06/68/66835.html

[33] Hermens HJ, Freriks B, Merletti R, Stegeman D, Blok J, Rau G, et al. SENIAM 8 European Recommendations for Surface ElectroMyoGraphy. Roessingh Research and Development b.v.; 1999.

[34] Ng JKF, Kippers V, Richardson CA. Muscle fibre orientation of abdominal muscles and suggested surface EMG electrode positions.—Abstract—Europe PMC. Electromyogr Clin Neurophysiol. 1998;38:51–8. 9532434

[35] Konrad P. The ABC of EMG: A Practical Introduction to Kinesiological Electromyography. Version 1.4. Scottsdale, Arizona: Noraxon U.S.A., Inc.; 2006.

[36] Sousa A, Tavares J. Surface electromyographic amplitude normalization methods: A review. In: Electromyography: New Developments, Procedures and Applications. 2012. p. 85–102.

[37] Winzeler-Merçay U, Mudie H. The nature of the effects of stroke on trunk flexor and extensor muscles during work and at rest. Disabil Rehabil. 2002 Jan 1;24(17):875–86. doi: 10.1080/09638280210142220 12519483

[38] R Core Team, editor. R: A Language and Environment for Statistical Computing. R Foundation for Statistical Computing, Vienna Austria; 2021.

[39] Bates D, Mächler M, Bolker B, Walker S. Fitting Linear Mixed-Effects Models Using lme4. J Stat Softw Vol 1 Issue 1 2015 [Internet]. 2015 Oct 7; Available from: https://www.jstatsoft.org/v067/i01

[40] Kuznetsova A, Brockhoff PB, Christensen RHB. lmerTest Package: Tests in Linear Mixed Effects Models. J Stat Softw Vol 1 Issue 13 2017 [Internet]. 2017 Dec 6; Available from: https://www.jstatsoft.org/v082/i13

[41] Revelle William. Procedures for Personality and Psychological Research [Internet]. Northwestern University, Evanston Illinois; 2021. Available from: https://CRAN.R-project.org/package=psych

[42] Lenth RV. emmeans: Estimated Marginal Means, aka Least-Squares Means [Internet]. 2021. Available from: https://CRAN.R-project.org/package=emmeans

[43] Wickham H. ggplot2: Elegant Graphics for Data Analysis [Internet]. Springer publishing New York; 2016. Available from: https://ggplot2.tidyverse.org

[44] Bae SH, Lee HG, Kim YE, Kim GY, Jung HW, Kim KY. Effects of Trunk Stabilization Exercises on Different Support Surfaces on the Cross-sectional Area of the Trunk Muscles and Balance Ability. J Phys Ther Sci. 2013/07/23 ed. 2013 Jun;25(6):741–5. doi: 10.1589/jpts.25.741 24259843 PMC3805005

[45] Duncan M. Muscle activity of the upper and lower rectus abdominis during exercises performed on and off a Swiss ball. J Bodyw Mov Ther. 2009 Oct 1;13(4):364–7. doi: 10.1016/j.jbmt.2008.11.008 19761961

[46] Van Criekinge T, Truijen S, Verbruggen C, Van de Venis L, Saeys W. The effect of trunk training on muscle thickness and muscle activity: a systematic review. Disabil Rehabil. 2019 Jul 17;41(15):1751–9. doi: 10.1080/09638288.2018.1445785 29502464

[47] Peach JP, Sutarno CG, McGill SM. Three-dimensional kinematics and trunk muscle myoelectric activity in the young lumbar spine: A database. Arch Phys Med Rehabil. 1998 Jun 1;79(6):663–9. doi: 10.1016/s0003-9993(98)90041-7 9630146

[48] Collins KC, Kennedy NC, Clark A, Pomeroy VM. Kinematic Components of the Reach-to-Target Movement After Stroke for Focused Rehabilitation Interventions: Systematic Review and Meta-Analysis. Front Neurol [Internet]. 2018;9. Available from: https://www.frontiersin.org/article/10.3389/fneur.2018.00472 29988530 10.3389/fneur.2018.00472 PMC6026634

[49] Cai S, Li G, Zhang X, Huang S, Zheng H, Ma K, et al. Detecting compensatory movements of stroke survivors using pressure distribution data and machine learning algorithms. J NeuroEngineering Rehabil. 2019 Nov 4;16(1):131. doi: 10.1186/s12984-019-0609-6 31684970 PMC6829931

[50] Farahpour N, Younesian H, Bahrpeyma F. Electromyographic activity of erector spinae and external oblique muscles during trunk lateral bending and axial rotation in patients with adolescent idiopathic scoliosis and healthy subjects. Clin Biomech. 2015 Jun 1;30(5):411–7. doi: 10.1016/j.clinbiomech.2015.03.018 25846325

[51] Hides J, Gilmore C, Stanton W, Bohlscheid E. Multifidus size and symmetry among chronic LBP and healthy asymptomatic subjects. Man Ther. 2008 Feb 1;13(1):43–9. doi: 10.1016/j.math.2006.07.017 17070721

[52] Kuriyama N, Ito H. Electromyographic Functional Analysis of the Lumbar Spinal Muscles with Low Back Pain. J Nippon Med Sch. 2005;72(3):165–73. doi: 10.1272/jnms.72.165 16046833

[53] Pereira LM, Marcucci FCI, de Oliveira Menacho M, Garanhani MR, Lavado EL, Cardoso JR. Electromyographic activity of selected trunk muscles in subjects with and without hemiparesis during therapeutic exercise. J Electromyogr Kinesiol. 2011 Apr 1;21(2):327–32. doi: 10.1016/j.jelekin.2010.10.003 21071243

[54] Castanharo R, Duarte M, McGill S. Corrective sitting strategies: An examination of muscle activity and spine loading. J Electromyogr Kinesiol. 2014 Feb 1;24(1):114–9. doi: 10.1016/j.jelekin.2013.11.001 24295543

[55] Kuiken TA, Lowery MM, Stoykov NS. The effect of subcutaneous fat on myoelectric signal amplitude and cross-talk. Prosthet Orthot Int [Internet]. 2003;27(1). Available from: https://journals.lww.com/poijournal/Fulltext/2003/27010/The_effect_of_subcutaneous_fat_on_myoelectric.8.aspx doi: 10.3109/03093640309167976 12812327

[56] Pappalardo A, Ciancio MR, Patti F. Is the basic trunk control recovery different between stroke patients with right and left hemiparesis? NeuroRehabilitation. 2014;35(2):215–20. doi: 10.3233/NRE-141109 24990016

[57] McGill SM, Yingling VR, Peach JP. Three-dimensional kinematics and trunk muscle myoelectric activity in the elderly spine–a database compared to young people. Clin Biomech. 1999 Jul 1;14(6):389–95. doi: 10.1016/s0268-0033(98)00111-9 10521620

[58] O’Sullivan P, Dankaerts W, Burnett A, Straker L, Bargon G, Moloney N, et al. Lumbopelvic Kinematics and Trunk Muscle Activity During Sitting on Stable and Unstable Surfaces. J Orthop Sports Phys Ther. 2006 Jan 1;36(1):19–25. doi: 10.2519/jospt.2006.36.1.19 16494070

[59] Perlmutter S, Lin F, Makhsous M. Quantitative analysis of static sitting posture in chronic stroke. Gait Posture. 2010 May;32(1):53–6. doi: 10.1016/j.gaitpost.2010.03.005 20399661

[60] Winter DA. Biomechanics and motor control of human movement. 4th edition. John Wiley & Sons Inc; 2009.

