# Supplementary material for: Surface electromyographic activity of trunk muscles during trunk control exercises for people after stroke; effect of a mobile and stable seat for rehabilitation
Source: PLoS One. 2022 Jul 29;17(7):e0272382. doi: 10.1371/journal.pone.0272382 (PMC9337656; doi:10.1371/journal.pone.0272382)

Supplementary material – interaction plots

For M. multifidi no interactions between the factors could be removed. In contrast, for M. erector spinae the factor side and the 4-way interaction could be removed. Therefore, interaction plots do not contain any information on the factor 'side'. In M. obliquus externus the 4-way interaction and interactions with side could be removed as well.

M. multifidi

S1 File. Interaction plot for predicted mean %STAT comparing the two groups for the multifidus muscles. CON = healthy participants, PAT = people after stroke, %STAT = percentage of maximal muscle activity relative to static sitting on the stable seat

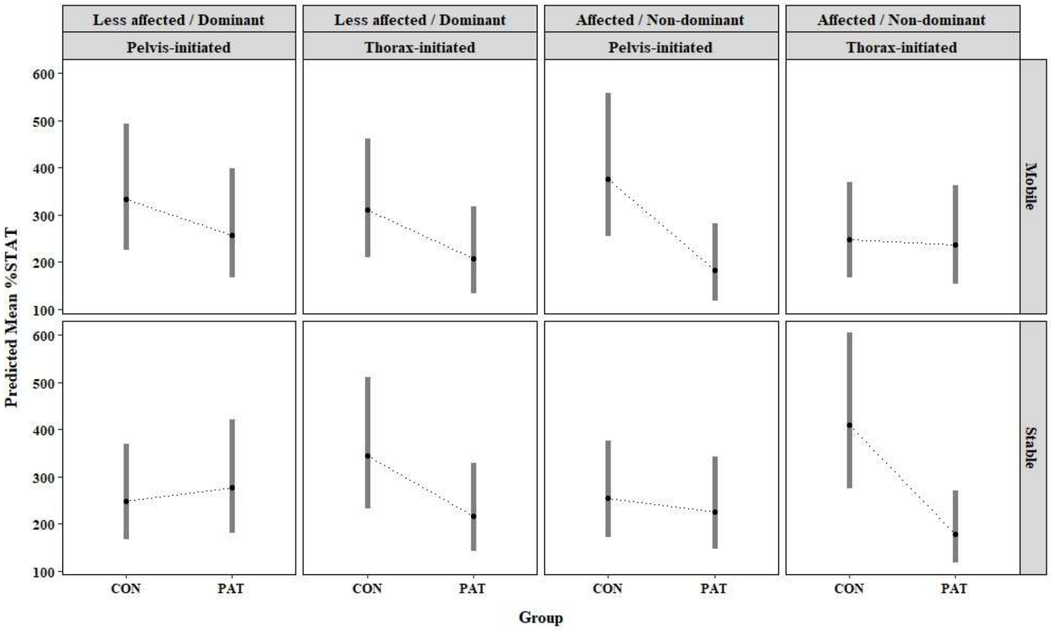

S1 File. Interaction plot for predicted mean %STAT comparing the mobile and the stable seat condition for the multifidus muscles. CON = healthy participants, PAT = people after stroke, %STAT = percentage of maximal muscle activity relative to static sitting on the stable seat

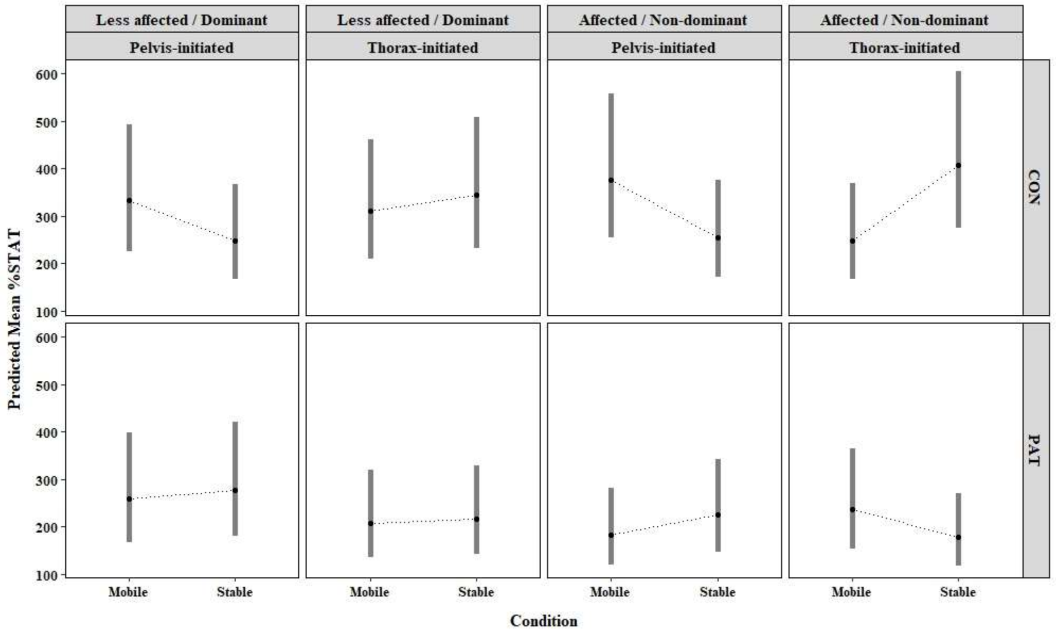

S1 File. Interaction plot for predicted mean %STAT comparing the sides for the multifidus muscles. CON = healthy participants, PAT = people after stroke, %STAT = percentage of maximal muscle activity relative to static sitting on the stable seat, dom/less aff = dominant/less affected, non-dom/aff = non-dominant/affected

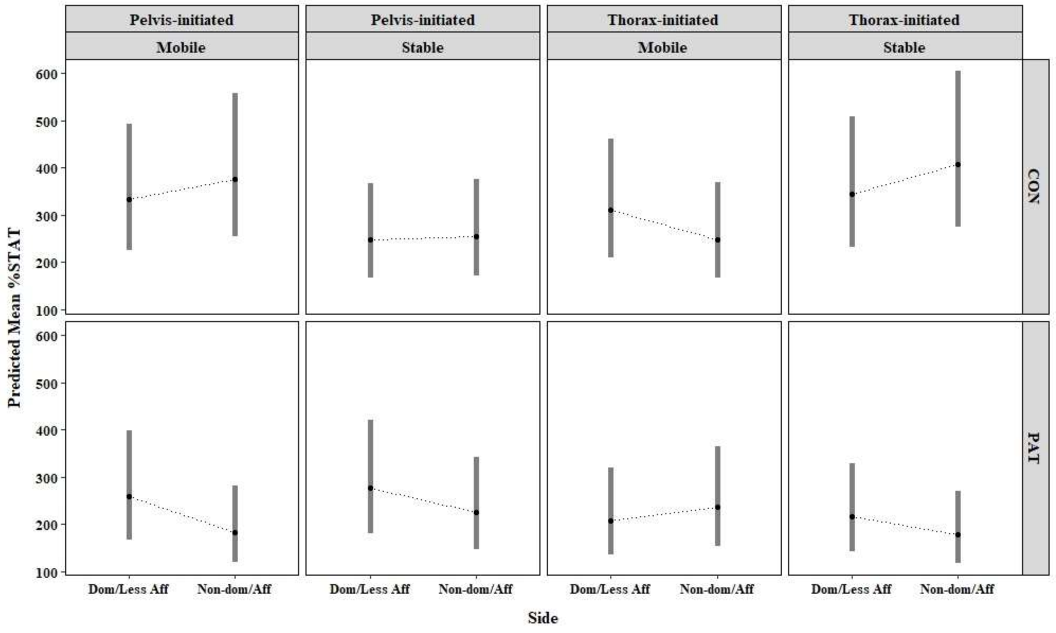

S1 File. Interaction plot for predicted mean %STAT comparing the thorax-initiated and the pelvis-initiated exercise for the multfidus muscles. CON = healthy participants, PAT = people after stroke, %STAT = percentage of maximal muscle activity relative to static sitting on the stable seat

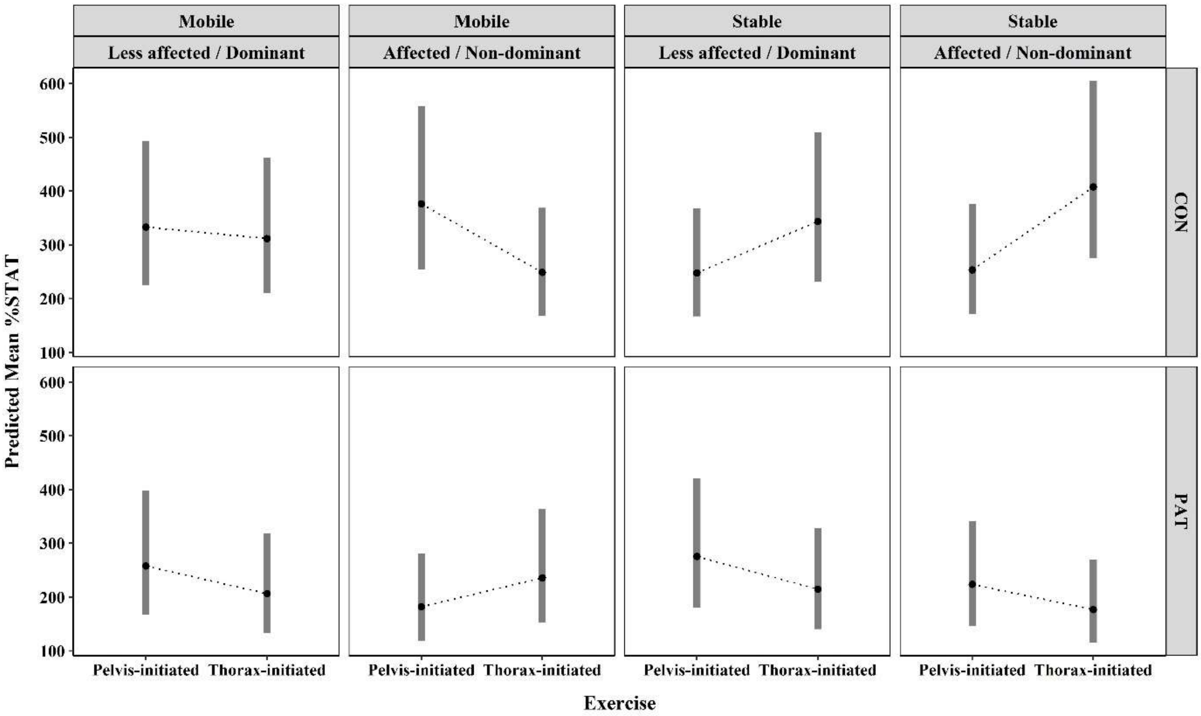

M. erector spinae

S1 File. Interaction plot for predicted mean %STAT comparing the two groups for the erector spinae. CON = healthy participants, PAT = people after stroke, %STAT = percentage of maximal muscle activity relative to static sitting on the stable seat

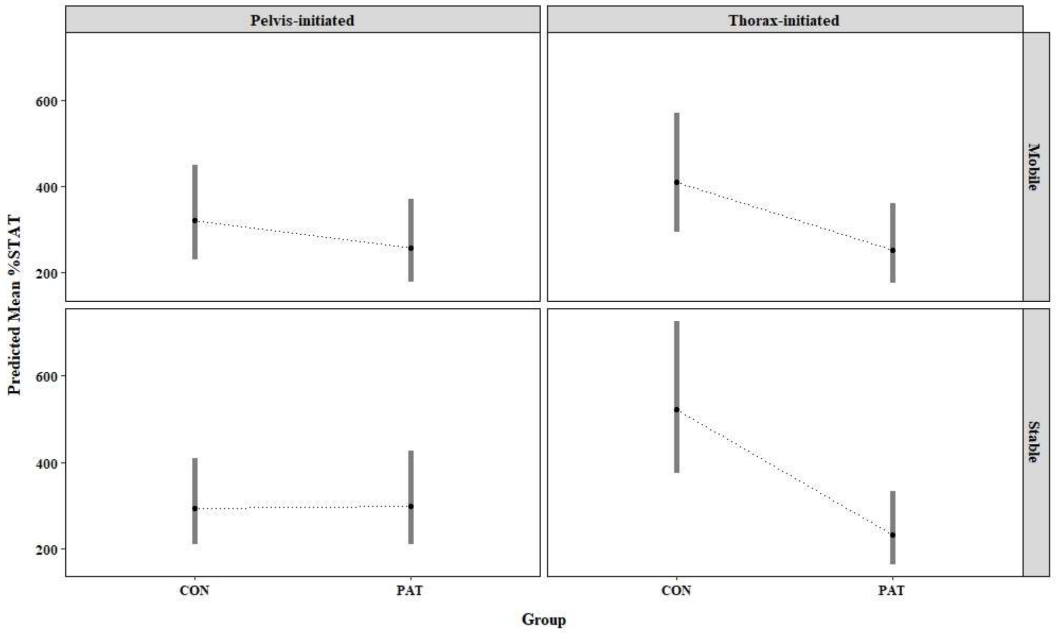

S1 File. Interaction plot for predicted mean %STAT comparing the mobile and the stable seat condition for the erector spinae. CON = healthy participants, PAT = people after stroke, %STAT = percentage of maximal muscle activity relative to static sitting on the stable seat

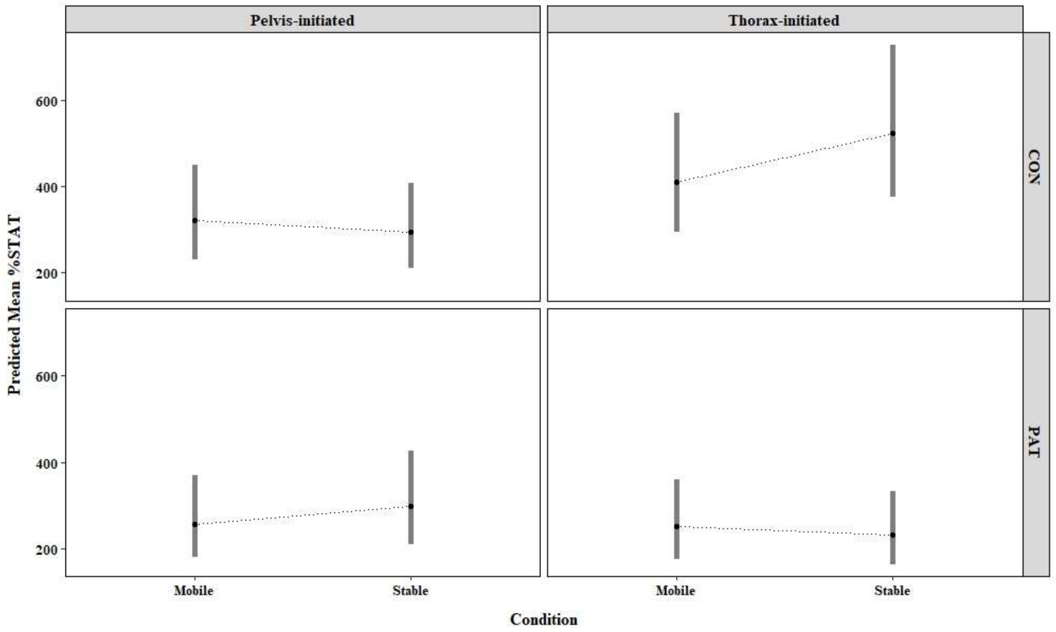

S1 File. Interaction plot for predicted mean %STAT comparing the pelvis- and the thorax-initiated exercise for the erector spinae. CON = healthy participants, PAT = people after stroke, %STAT = percentage of maximal muscle activity relative to static sitting on the stable seat

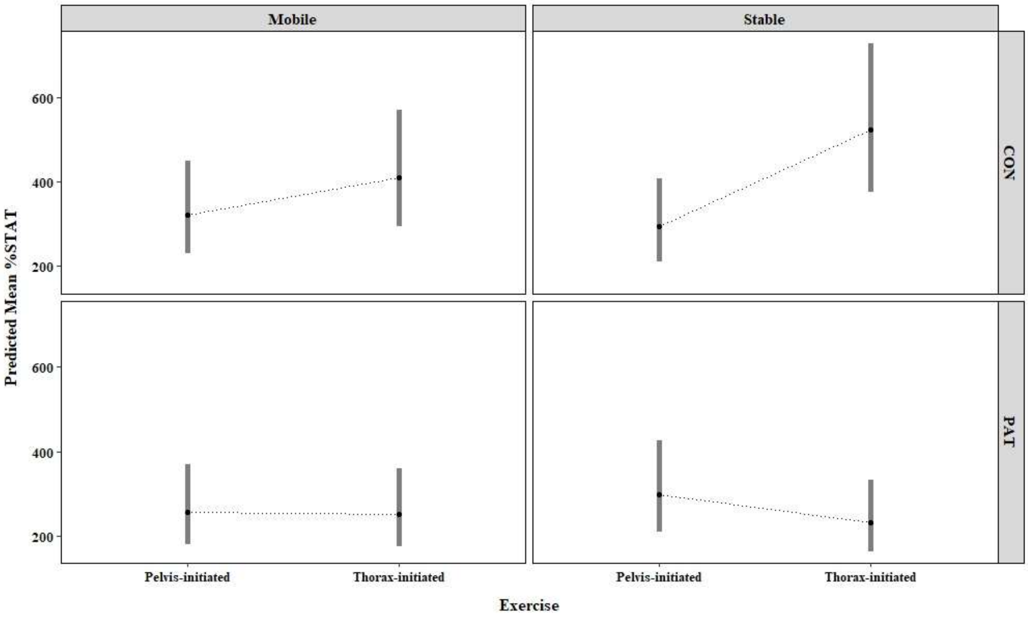

M. obliquus externus

S1 File. Interaction plot for predicted mean %STAT comparing the two groups for the obliquus externus. CON = healthy participants, PAT = people after stroke, %STAT = percentage of maximal muscle activity relative to static sitting on the stable seat

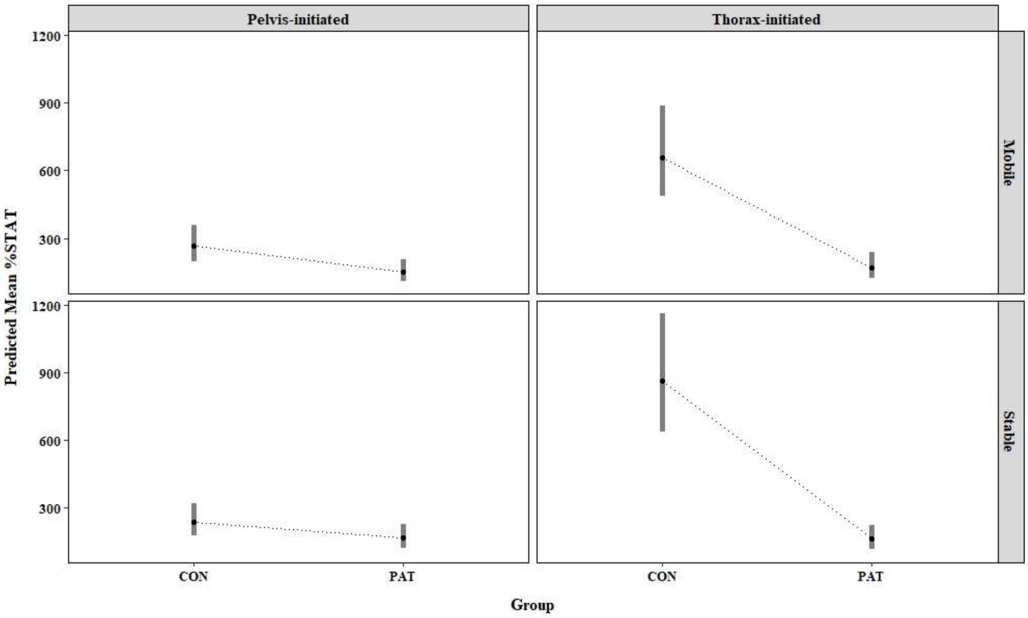

S1 File. Interaction plot for predicted mean %STAT comparing the mobile and the stable seat condition for the obliquus externus. CON = healthy participants, PAT = people after stroke, %STAT = percentage of maximal muscle activity relative to static sitting on the stable seat

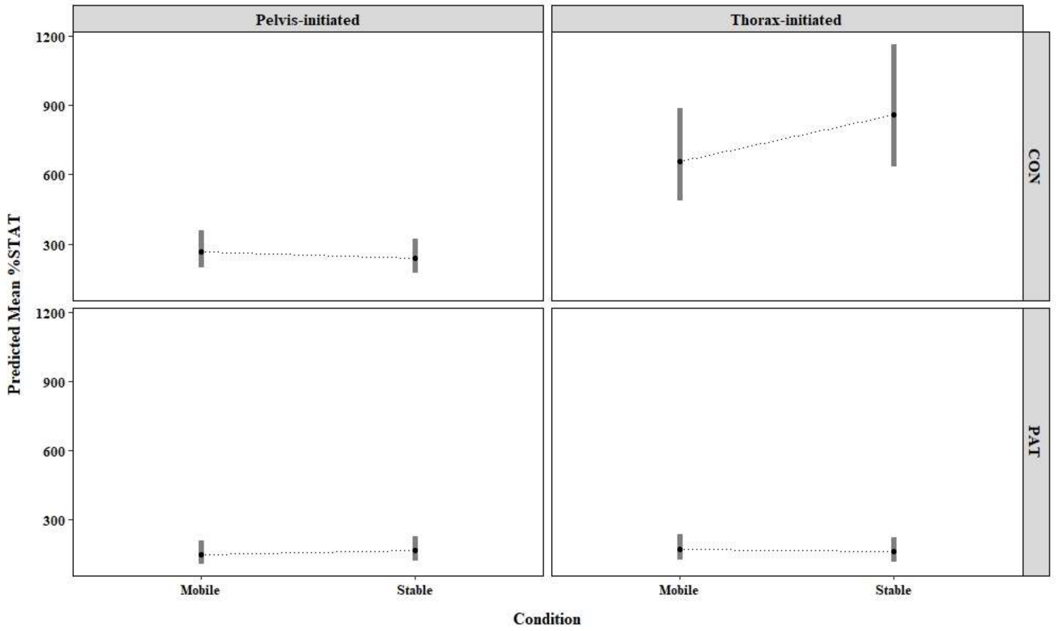

S1 File. Interaction plot for predicted mean %STAT comparing the sides for the obliquus externus. CON = healthy participants, PAT = people after stroke, %STAT = percentage of maximal muscle activity relative to static sitting on the stable seat, dom/less aff = dominant/less affected, non-dom/aff = non-dominant/affected

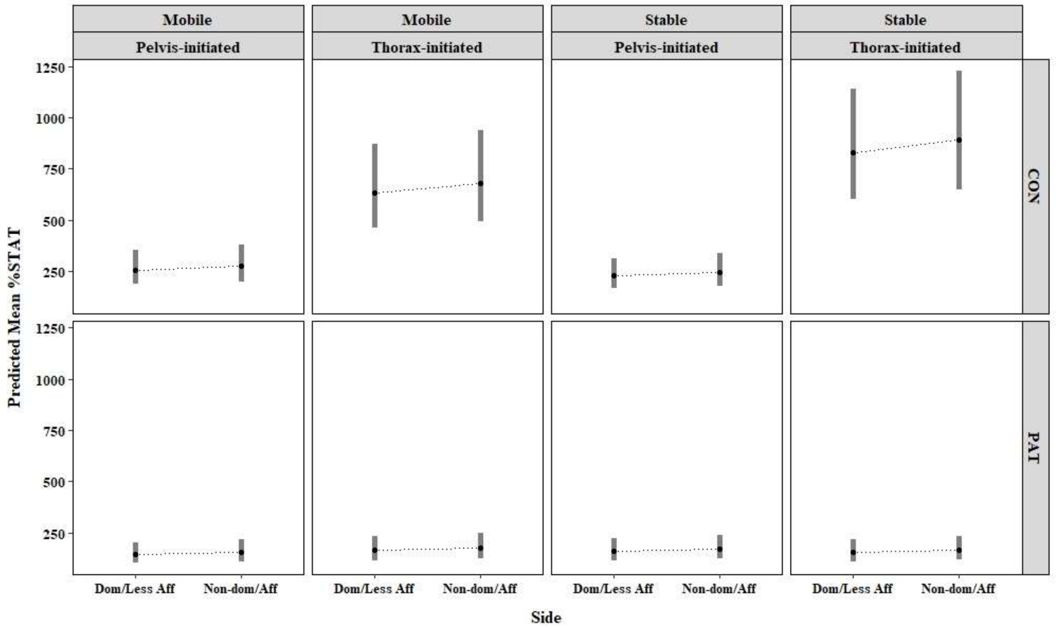

S1 File. Interaction plot for predicted mean %STAT comparing the pelvis- and the thorax-initiated exercise for the obliquus externus. CON = healthy participants, PAT = people after stroke, %STAT = percentage of maximal muscle activity relative to static sitting on the stable seat

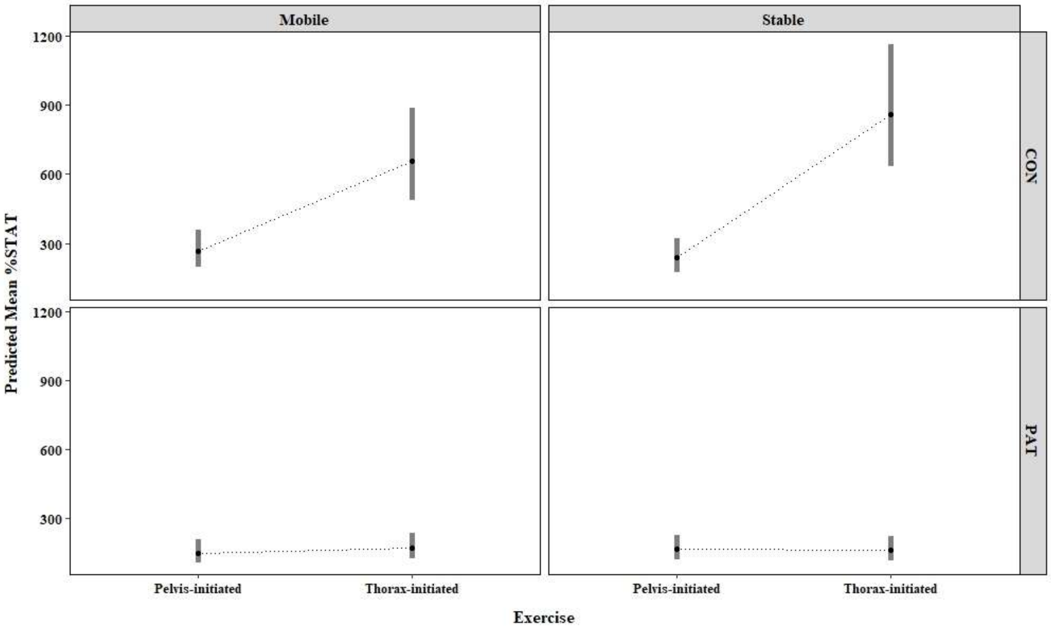

Supplement: S1 File — (PDF) [file pone.0272382.s001.pdf]
