# Supplementary material for: Surface electromyographic activity of trunk muscles during trunk control exercises for people after stroke; effect of a mobile and stable seat for rehabilitation
Source: PLoS One. 2022 Jul 29;17(7):e0272382. doi: 10.1371/journal.pone.0272382 (PMC9337656; doi:10.1371/journal.pone.0272382)
Supplement: S2 File — (DOCX) [file pone.0272382.s002.docx]

Supplementary material – predicted and descriptive sEMG data

S2 File. Predicted sEMG means for the three muscles multifidus, erector spinae, and obliquus externus

|  | Multifidus | | | | | | Erector spinae | | | | | | | | Obliquus externus | | | | | | |
| --- | --- | --- | --- | --- | --- | --- | --- | --- | --- | --- | --- | --- | --- | --- | --- | --- | --- | --- | --- | --- | --- |
| Seat | Side | Group | Exercise | Response  %STAT | 95% CL | | | Seat | Side | Group | Exercise | Response | 95% CL | | Seat | Side | Group | Exercise | Response  %STAT | 95% CL | |
|  |  |  |  |  | Lower | Upper | |  |  |  |  | %STAT | Lower | Upper |  |  |  |  |  | Upper | Lower |
| Mobile | Less affected / dominant | CON | Pelvis | 333.04 | 224.63 | 493.75 | | Mobile | Less affected / dominant | CON | Pelvis | 321.79 | 231.19 | 447.89 | Mobile | Less affected / dominant | CON | Pelvis | 255.96 | 186.18 | 351.89 |
|  |  |  | Thorax | 311.74 | 210.27 | 462.18 | |  |  |  | Thorax | 409.82 | 294.44 | 570.42 |  |  |  | Thorax | 632.86 | 460.33 | 870.05 |
|  |  | PAT | Pelvis | 257.89 | 167.26 | 397.64 | |  |  | PAT | Pelvis | 257.30 | 179.27 | 369.29 |  |  | PAT | Pelvis | 143.62 | 102.27 | 201.70 |
|  |  |  | Thorax | 206.47 | 133.90 | 318.35 | |  |  |  | Thorax | 251.33 | 175.11 | 360.73 |  |  |  | Thorax | 164.72 | 117.29 | 231.33 |
|  | Affected / non-dominant | CON | Pelvis | 376.27 | 253.80 | 557.85 | |  | Affected / non-dominant | CON | Pelvis | 321.79 | 231.19 | 447.89 |  | Affected / non-dominant | CON | Pelvis | 275.31 | 200.26 | 378.50 |
|  |  |  | Thorax | 248.92 | 167.90 | 369.04 | |  |  |  | Thorax | 409.82 | 294.44 | 570.42 |  |  | PAT | Thorax | 680.72 | 495.14 | 935.85 |
|  |  | PAT | Pelvis | 182.12 | 118.12 | 280.81 | |  |  | PAT | Pelvis | 257.30 | 179.27 | 369.29 |  |  | CON | Pelvis | 154.48 | 109.58 | 217.78 |
|  |  |  | Thorax | 235.78 | 152.92 | 363.55 | |  |  |  | Thorax | 251.33 | 175.11 | 360.73 |  |  | PAT | Thorax | 177.18 | 125.68 | 249.77 |
| Stable | Less affected / dominant | CON | Pelvis | 247.84 | 167.17 | 367.45 | | Stable | Less affected / dominant | CON | Pelvis | 293.29 | 210.71 | 408.22 | Stable | Less affected / dominant | CON | Pelvis | 227.93 | 165.79 | 313.35 |
|  |  |  | Thorax | 343.67 | 231.80 | 509.51 | |  |  |  | Thorax | 522.33 | 375.27 | 727.02 |  |  | PAT | Thorax | 829.87 | 603.63 | 1140.90 |
|  |  | PAT | Pelvis | 275.61 | 180.55 | 420.72 | |  |  | PAT | Pelvis | 297.78 | 208.76 | 424.76 |  |  | CON | Pelvis | 159.57 | 114.66 | 222.08 |
|  |  |  | Thorax | 214.71 | 140.65 | 327.75 | |  |  |  | Thorax | 232.71 | 163.14 | 331.95 |  |  | PAT | Thorax | 155.96 | 112.06 | 217.05 |
|  | Affected / non-dominant | CON | Pelvis | 253.53 | 171.01 | 375.87 | |  | Affected / non-dominant | CON | Pelvis | 293.29 | 210.71 | 408.22 |  | Affected / non-dominant | CON | Pelvis | 245.16 | 178.33 | 337.05 |
|  |  |  | Thorax | 408.07 | 275.24 | 604.99 | |  |  |  | Thorax | 522.33 | 375.27 | 727.02 |  |  | PAT | Thorax | 892.63 | 649.28 | 1227.19 |
|  |  | PAT | Pelvis | 223.98 | 146.72 | 341.90 | |  |  | PAT | Pelvis | 297.78 | 208.76 | 424.76 |  |  | CON | Pelvis | 171.64 | 122.87 | 239.78 |
|  |  |  | Thorax | 176.85 | 115.85 | 269.96 | |  |  |  | Thorax | 232.71 | 163.14 | 331.95 |  |  | PAT | Thorax | 167.76 | 120.08 | 234.35 |

%STAT = percentage of maximal muscle activity relative to static sitting on the stable seat, CL = confidence level, CON = healthy participants, PAT = people after stroke; predicted means identical in both sides of erector spinae due to removed covariate

S2 File. Descriptive sEMG means and standard deviation of each subgroup for the pelvis-initiated lateral flexion in M. multifidi.

| Seat | Side | Group | N | Mean %STAT | SD |
| --- | --- | --- | --- | --- | --- |
| Mobile | Less affected/dominant | CON | 15 | 420.56 | 304.85 |
|  |  | PAT | 12 | 340.27 | 247.08 |
|  | Affected/non-dominant | CON | 15 | 609.81 | 686.94 |
|  |  | PAT | 12 | 229.11 | 163.47 |
| Stable | Less affected/dominant | CON | 15 | 363.74 | 477.18 |
|  |  | PAT | 13 | 352.45 | 309.52 |
|  | Affected/non-dominant | CON | 15 | 482.28 | 742.15 |
|  |  | PAT | 13 | 234.40 | 72.92 |

CON = healthy participants, PAT = people after stroke, N = number of measurements, %STAT = percentage of maximal muscle activity relative to static sitting on the stable seat, SD = standard deviation

S2 File. Descriptive sEMG means and standard deviation of each subgroup of the thorax-initiated lateral flexion in M. multifidi.

| Seat | Side | Group | N | Mean %STAT | SD |
| --- | --- | --- | --- | --- | --- |
| Mobile | Less affected/dominant | CON | 15 | 388.34 | 305.29 |
|  |  | PAT | 12 | 303.09 | 287.42 |
|  | Affected/non-dominant | CON | 15 | 320.94 | 226.42 |
|  |  | PAT | 12 | 284.30 | 188.99 |
| Stable | Less affected/dominant | CON | 15 | 430.92 | 315.27 |
|  |  | PAT | 13 | 323.20 | 310.16 |
|  | Affected/non-dominant | CON | 15 | 459.97 | 219.83 |
|  |  | PAT | 13 | 231.46 | 196.08 |

CON = healthy participants, PAT = people after stroke, N = number of measurements, %STAT = percentage of maximal muscle activity relative to static sitting on the stable seat, SD = standard deviation

S2 File. Descriptive sEMG means and standard deviation for each subgroup for the pelvis-initiated lateral flexion of the M. erector spinae.

| Seat | Side | Group | N | Mean %STAT | SD |
| --- | --- | --- | --- | --- | --- |
| Mobile | Less affected/dominant | CON | 15 | 483.94 | 437.90 |
|  |  | PAT | 12 | 312.07 | 222.40 |
|  | Affected/non-dominant | CON | 15 | 438.22 | 454.74 |
|  |  | PAT | 12 | 344.53 | 284.28 |
| Stable | Less affected/dominant | CON | 15 | 541.93 | 868.98 |
|  |  | PAT | 13 | 297.75 | 135.13 |
|  | Affected/non-dominant | CON | 15 | 315.99 | 213.09 |
|  |  | PAT | 13 | 366.57 | 200.25 |

CON = healthy participants, PAT = people after stroke, N = number of measurements, %STAT = percentage of maximal muscle activity relative to static sitting on the stable seat, SD = standard deviation

S2 File. Descriptive sEMG means and standard deviation of each subgroup for the thorax-initiated lateral flexion of the M. erector spinae.

| Seat | Side | Group | N | Mean %STAT | SD |
| --- | --- | --- | --- | --- | --- |
| Mobile | Less affected/dominant | CON | 15 | 659.67 | 584.65 |
|  |  | PAT | 12 | 280.16 | 205.50 |
|  | Affected/non-dominant | CON | 15 | 416.68 | 307.73 |
|  |  | PAT | 12 | 364.79 | 272.44 |
| Stable | Less affected/dominant | CON | 15 | 745.23 | 760.17 |
|  |  | PAT | 13 | 287.44 | 225.71 |
|  | Affected/non-dominant | CON | 15 | 673.55 | 610.88 |
|  |  | PAT | 13 | 346.68 | 289.75 |

CON = healthy participants, PAT = people after stroke, N = number of measurements, %STAT = percentage of maximal muscle activity relative to static sitting on the stable seat, SD = standard deviation

S2 File. Descriptive means and standard deviation for each subgroup for the pelvis-initiated lateral flexion in M. obliquus externus.

| Seat | Side | Group | N | Mean %STAT | SD |
| --- | --- | --- | --- | --- | --- |
| Mobile | Less affected/dominant | CON | 14 | 361.23 | 380.97 |
|  |  | PAT | 12 | 165.76 | 113.11 |
|  | Affected/non-dominant | CON | 14 | 343.43 | 185.49 |
|  |  | PAT | 12 | 180.53 | 83.23 |
| Stable | Less affected/dominant | CON | 14 | 279.94 | 236.51 |
|  |  | PAT | 12 | 195.14 | 104.46 |
|  | Affected/non-dominant | CON | 14 | 292.27 | 159.25 |
|  |  | PAT | 13 | 195.24 | 126.89 |

CON = healthy participants, PAT = people after stroke, N = number of measurements, %STAT = percentage of maximal muscle activity relative to static sitting on the stable seat, SD = standard deviation

S2 File. Descriptive sEMG means and standard deviation of each subgroup for thorax-initiated lateral flexion for M. obliquus externus.

| Seat | Side | Group | N | Mean %STAT | SD |
| --- | --- | --- | --- | --- | --- |
| Mobile | Less affected/dominant | CON | 14 | 910.16 | 736.45 |
|  |  | PAT | 12 | 193.72 | 128.28 |
|  | Affected/non-dominant | CON | 14 | 789.95 | 734.82 |
|  |  | PAT | 12 | 189.95 | 82.38 |
| Stable | Less affected/dominant | CON | 14 | 1065.89 | 806.07 |
|  |  | PAT | 12 | 199.64 | 159.18 |
|  | Affected/non-dominant | CON | 14 | 1131.17 | 783.80 |
|  |  | PAT | 13 | 193.54 | 92.95 |

CON = healthy participants, PAT = people after stroke, N = number of measurements, %STAT = percentage of maximal muscle activity relative to static sitting on the stable seat, SD = standard deviation
